# Effects of physical activity and resilience on emotional and behavioral problems in Chinese adolescent: a chained mediation model

**DOI:** 10.3389/fpsyg.2025.1486949

**Published:** 2025-07-07

**Authors:** YiJie Shi, Wei Zhao, Mariusz Lipowski

**Affiliations:** ^1^Faculty of Physical Culture, Gdansk University of Physical Education and Sport, Gdańsk, Poland; ^2^College of Physical Education, Northwest Minzu University, Lanzhou, Gansu, China; ^3^Faculty of Social and Humanities, WSB Merito University in Gdańsk, Gdańsk, Poland

**Keywords:** physical activity, parenting style, emotional and behavioral problems, resilience, Chinese adolescents

## Abstract

**Background:**

Adolescent emotional and behavioral problems (EBPs) represent a global health challenge, affecting approximately 970 million individuals worldwide (2019). Adolescence constitutes a critical developmental period characterized by heightened vulnerability to body image concerns, interpersonal stressors, and mental health risks. While parenting styles and physical activity (PA) independently influence adolescent psychological outcomes, their synergistic effects through psychological resilience remain inadequately explored, particularly regarding cultural nuances in Chinese parenting practices (e.g., dual interpretations of overprotection) and chained mediation pathways (parenting → PA → resilience → EBPs).

**Objective:**

This study examined the chained mediating roles of physical activity and psychological resilience in the relationship between multidimensional parenting styles (emotional warmth, rejection, overprotection) and EBPs among Chinese adolescents, testing pathways informed by family systems theory and bioecological frameworks.

**Participants and setting:**

A sample of 503 adolescents (51.09% male; SD = 1.41) was recruited through three-stage cluster sampling across Zhuhai, Guangdong, China. Participants were purposively selected from transition grades: elementary (Grades 5–6, ages 10–12) and junior high schools (Grades 7–9, ages 13–15) across 8 institutions (4 key + 4 general schools). Data collection occurred between April-December 2024, with 89.83% retention after excluding incomplete/invalid responses (logical inconsistencies, accelerometer data <3 valid days). No significant demographic differences existed between retained and excluded cases (*p* > 0.05).

**Methods:**

Validated instruments measured: EBPs (Strengths and Difficulties Questionnaire, α = 0.79), parenting styles (EMBU short form: Rejection α = 0.890; Overprotection α = 0.824; Emotional Warmth α = 0.910), resilience (Connor-Davidson Resilience Scale, α = .970), and PA (IPAQ-SF MET-min/week, α = 0.936). Confirmatory factor analysis established measurement models (RMSEA = 0.026, CFI = 0.971, TLI = 0.970). Structural equation modeling with 5,000 bootstrap resamples (PROCESS Model 6) tested three mediation pathways: (1) Parenting → PA → EBPs; (2) Parenting → Resilience → EBPs; (3) Parenting → PA → Resilience → EBPs.

**Results:**

Emotional warmth significantly predicted reduced EBPs through increased PA (*B* = 0.862, *p* < 0.001) and resilience (*B* = 0.571, *p* < 0.001), with PA demonstrating the strongest independent mediation (effect = 0.544, 95% CI [0.460, 0.629]). Rejection exacerbated EBPs by suppressing PA (*B* = −0.318, *p* < 0.001) and resilience (*B* = −0.294, *p* < 0.001; total indirect effect = 0.412, CI [0.332, 0.503]). Overprotection influenced EBPs solely through reduced PA (effect = 0.102, CI [0.058, 0.158]), showing no significant resilience association. The full chained mediation (parenting → PA → resilience → EBPs) was significant (effect = 0.075, CI [0.006, 0.145]), accounting for 24.23% of the total effect.

## 1 Introduction

Adolescents frequently encounter stressful events, such as body image concerns related to physical development (Dakanalis et al., 2016) and interpersonal relationship stress (Byrne et al., 2007). Compared to adults, adolescents lack social experience and strategies to cope with stress, making them more susceptible, and high risk for mental health, such as depression (Young and Dietrich, 2015), anxiety (Anyan and Hjemdal, 2016), and addictive behavior (Li et al., 2016). Emotional and behavioral problems (EBPs) are prevalent mental health issues, with an estimated 970 million people affected globally in 2019 (Global Burden of Disease Study, 2019).

Psychological resilience, an essential protective factor, refers to the ability to adapt positively to adversity or trauma (Luthar et al., 2003). Resilience is a positive trait that aids in overcoming difficulties and facilitating mental recovery. Studies based on the resilience theory considered resilience as a protective factor for adversity coping (Carrasco et al., 2015). Stressor assessment, emotional response metacognition, and coping strategy selection could influence the mental health of individuals (Fletcher and Scott, 2010). Resilience can enhance adolescents’ school adaptability (Zhang et al., 2019) and wellbeing (Satici, 2016), and alleviate their depression and anxiety (Anyan and Hjemdal, 2016). Thus, it is worthy to investigate the environmental factors that promote resilience in adolescents and how the factors work.

Physical activity can significantly enhance psychological resilience and mental health of adolescents. It mitigates challenges across psychological, social, and physical domains and enhances positive psychological properties such as life satisfaction and happiness (Kim, 2009). Additionally, physical activity improves physical health (Lim and Nam, 2008) and generates positive psychological effects (Paluska and Schwenk, 2000). It is effective in reducing stress, anxiety, and depression (Buckworth et al., 2013; Hassmén et al., 2000). There is strong evidence that physical activity plays a major role in promoting physical health and psychological wellbeing. Motivation, particularly intrinsic motivation (such as enjoyment and competence) and externally regulated motivation (such as social recognition and compliance with expectations), has been identified as a robust predictor of sustained participation in physical activity (Morris and Roychowdhury, 2020a; Ryan and Deci, 2000). For adolescents, organized physical activities (such as team sports and school-based programs) are uniquely appealing due to their structured social environment, which provides opportunities for peer bonding, coach mentorship, and progressive skill development (Belcher et al., 2021). These features align with adolescents’ developmental needs for autonomy, social belonging, and identity exploration (Eccles and Gootman, 2002), thus enhancing engagement and adherence compared to unstructured activities. Furthermore, physical activity contributes to emotional health, self-confidence, and social skills development (Lee et al., 2011) and enhances positive emotions (Delvecchio et al., 2020). For adolescents, organized physical activities are attractive and serve as an effective intervention for mental health (Belcher et al., 2021), as well as psychological resilience. Guo and Liang (2023) reported a positive correlation between physical activities and adolescent psychological resilience. The mechanisms through which physical activity enhances psychological resilience could be explained through multidimensional pathways. Firstly, at the neurobiological level, regular exercise stimulates the release of neurotransmitters such as serotonin and endorphins while reducing cortisol levels (Buckworth et al., 2013). These biochemical changes improve prefrontal cortex-mediated emotional regulation and mitigate stress responses (Delvecchio et al., 2020; Kim, 2009). Secondly, psychosocial mechanisms operate through two channels, the achievement of physical goals (such as endurance training or skill mastery) enhances self-efficacy beliefs in overcoming challenges (Lee et al., 2011; Morris and Roychowdhury, 2020b), and organized activities (such as team sports) foster peer interactions and coach-guided support, thereby strengthening social belonging, as a core protective factor for resilience (Belcher et al., 2021; Ho et al., 2015). Additionally, behavioral regulation mechanisms involve distraction from rumination through exercise engagement (Hassmén et al., 2000), coupled with the development of self-discipline and adaptive coping strategies through habitual training (Andermo et al., 2020). School-based physical activity interventions, which systematically integrate these mechanisms, have demonstrated significant efficacy in promoting adolescent resilience (Andermo et al., 2020). Another research on Hong Kong adolescents revealed a significant correlation between the level of physical activity and wellbeing and resilience, suggesting resilience improves mental health (Ho et al., 2015). Additionally, a systematic review and meta-analysis by Andermo et al. (2020) revealed significant effects of school-based physical activities on enhancing adolescent resilience, promoting positive mental health, increasing wellbeing, and reducing anxiety. This underscores the vital role of school-based physical activities in enhancing the mental health of children and adolescents.

From the perspective of the psychological resilience framework, the environmental background is considered as a significant factor. Parenting plays a critical role in the development of adolescent resilience (Kumpfer, 2002) and encompasses a range of complex behaviors, methods, and attitudes employed by parents in raising and educating their children. The method of parenting is closely related to adolescent psychological development outcomes within different cultural backgrounds. Positive parenting provides emotional and behavioral support to adolescents and aids in building psychological resilience (Tian et al., 2018). superior parenting method was revealed to enhance the positive psychological resources of child and correctly coordinate factors positively correlated with psychological resilience (Kwok et al., 2019). Conversely, negative parenting methods are associated with higher risks of anxiety, depression, and aggressive behavior in adolescents (Smokowski et al., 2015). For instance, overprotection (Qu et al., 2024), emotional coldness, or rejection could potentially lead to depression, anxiety, addiction, and antagonistic psychology (Cui and Lan, 2020), thereby hindering the development of resilience (Liu et al., 2024). Studies have shown that authoritative parenting, characterized by emotional support, is positively correlated with healthy adaptability and psychosocial skills in young children. Authoritative parenting styles can prevent the internalization of symptoms such as anxiety and depression (Adubale, 2017; Delvecchio et al., 2020). In contrast, negative parenting styles, such as those of authoritarian parents, diminish children’s autonomy by reinforcing obedience and appearing less emotionally responsive (Parke, 2004; Shaw and Starr, 2019). Indulgent parents fail to create an environment conducive to developing children’s self-control due to their lenient rules. As a result, dictatorial and indulgent educational approaches are not beneficial to children’s psychological health development. While authoritative parenting enhances resilience through emotional support (Tian et al., 2018), authoritarian or permissive styles may undermine adolescents’ psychological health by fostering dependency or impulsivity (Parke, 2004). Extended research indicates that different parenting styles have varying effects on adolescents’ psychological states (Ding et al., 2022), with recent evidence highlighting nuanced differences in responsive parenting behaviors between mothers and fathers (Anikiej-Wiczenbach et al., 2024). These variations highlight the need to consider cultural and contextual factors in parenting interventions. For example, co-parenting strategies that align with authoritative principles can mitigate the risks of inconsistent discipline (Feinberg et al., 2007). Moreover, co-parenting, a unique family structure, has a significant impact on children’s development. Many studies suggest that differences in parental upbringing determine preschool children’s problem behaviors (Deal et al., 1989), and positive co-parenting can help children reduce problem behaviors (Feinberg et al., 2007; Schoppe et al., 2001).

According to the Family Systems Theory, an individual’s healthy development depends on the wellbeing of the family system. The parent-child relationship is a crucial subsystem within this system, and parenting styles are essential components (Parke, 2004). Parenting styles refer to the strategies parents use in raising children, encompassing psychological constructs such as emotional warmth, rejection, and overprotection (Darling and Steinberg, 1993). Positive and negative parenting styles have different effects on adolescents’ EBPs. Specifically, parental emotional warmth has a positive influence on emotions and behaviors (Tobe et al., 2019). It is positively correlated with adolescents’ social skills and peer attachment (Hosokawa and Katsura, 2017; Zou et al., 2023). Authoritative parents can provide emotional support and are related to young children’s healthy adaptability and psychosocial competencies. In addition, authoritative parenting styles can prevent the internalization of symptoms such as anxiety and depression (Adubale, 2017; Delvecchio et al., 2020). Whereas, negative parenting styles, such as low emotional warmth, overprotection, or rejection, may be strongly associated with depression, anxiety, addiction, and hostility (Cui and Lan, 2020). Authoritarian parents limit children’s autonomy by fostering a strong sense of obedience while offering little emotional response to their needs, and indulgent parents lack the rule-providing atmosphere necessary for children to develop self-control (Francis et al., 2021). Therefore, authoritarian and indulgent parenting styles can be detrimental to the psychological development of children.

Many researchers have investigated risk factors affecting adolescents’ EBPs, emphasizing family roles (Cox and Paley, 1997). The bioecological theory of Bronfenbrenner positions the family as a microsystem directly shaping emotional and behavioral outcomes through parenting styles (Tudge et al., 2009; Qu et al., 2024). Authoritative responsive yet structured parenting reduces internalizing behaviors by fostering secure attachment and self-regulation (Luebbe et al., 2018), while authoritarian styles, such as harsh control, correlate with externalizing behaviors like aggression (Lansford et al., 2014).

Indirect pathways are evident in outer systems: parental work stress ecosystem increases family conflict, elevating EBPs through disrupted monitoring (Crouter and Bumpus, 2001). Cultural norms macrosystem moderate these effects; Authoritarian practices show weaker harm in collectivist contexts (Steinberg, 2015). Longitudinal evidence links chronic harsh parenting to HPA-axis dysregulation and EBPs (McLoyd et al., 2007), while multi-system interventions including parent training and school support significantly reduce EBPs (Webster-Stratton and Reid, 2010).

Several researchers reviewed the association between physical activities and youth mental health. Physical activities significantly benefit adolescents by promoting physical health, improving mental states, enhancing cognitive function, reducing anxiety and depression, and strengthening self-concept, thereby elevating overall mental health (Ferreira-Vorkapic et al., 2015). Biddle and Asare (2011) demonstrated that physical activities have a positive influence on the mental health of children and adolescents. Physical activity enhances cognitive function through neurobiological mechanisms, primarily by increasing brain-derived neurotrophic factor (BDNF) to stimulate hippocampal neurogenesis and prefrontal cortex plasticity, thereby improving memory and executive function (Erickson et al., 2011; Hillman et al., 2009). For example, 6 months of aerobic exercise boosts hippocampal volume in older adults, correlating with better spatial memory, while 20 min of brisk walking acutely enhances children’s attention by 10%. Chronic exercise further strengthens neural networks, mitigating age-related cognitive decline. Although physical activity indirectly alleviates depression-related cognitive impairments via serotonin and BDNF upregulation (Kandola et al., 2019), its effects on anxiety are context-dependent, while reducing trait anxiety through amygdala modulation, acute anxiety benefits are modest (Biddle and Asare, 2011), likely due to individual variability in stress responses. Rodriguez-Ayllon et al. (2019) analyzed the impact of physical activity interventions, including randomized and non-randomized controlled trials, on the mental health conditions of adolescents. Consistent with observational evidence from longitudinal studies (such as 15% lower depression risk per 30-min daily activity, Kandola et al., 2020) and cross-sectional data [stronger effects in team sports (*d* = 0.51) vs. individual exercises (*d* = 0.29)], structured physical activity interventions demonstrate clinically meaningful improvements in adolescent mental health. A meta-analysis of 38 RCTs (Rodriguez-Ayllon et al., 2019) revealed moderate effect sizes (*g* = 0.47, 95% CI 0.32–0.62), with depression reduction (SMD = –0.53), anxiety alleviation (SMD = -0.41), and self-esteem enhancement (SMD = 0.37) being most pronounced when interventions lasted ≥ 8 weeks and incorporated social interaction components such as group sports vs. solo training (*p* < 0.05).

While physical activity has been shown to positively impact adult mental health (Fox, 1999; Scully et al., 1998) and prevent the onset of mental health disorders (Cooney et al., 2013; Paluska and Schwenk, 2000), research on youth populations remains limited and methodologically inconsistent. Key limitations include: (1) heterogeneous measurement approaches, where studies vary in defining physical activity (such as self-reported frequency vs. accelerometer-measured intensity) and mental health outcomes (such as a narrow focus on self-esteem; Valois et al., 2008 vs. comprehensive assessments of emotional/behavioral problems). Self-reports, used in 78% of studies (Opdal et al., 2019), are prone to recall bias, whereas objective tools like accelerometers (Toseeb et al., 2014) provide valid data on activity duration and intensity; (2) unclear mediating pathways. Although theoretical models propose that physical activity and psychological resilience mediate the link between parenting styles and adolescent mental health (Van Dijk et al., 2016), empirical support is scarce. For example, Biddle et al. (2019) found that adolescents with authoritative parents engaged in 23% more moderate-to-vigorous physical activity (*p* = 0.01), which partially mediated reduced internalizing symptoms (β = −0.17, *p* < 0.05) through enhanced resilience. However, replication with multi-informant designs and biomarker validation is needed to confirm these pathways.

In the present study, there is a meaningful correlation between EBPs, parenting methods, and psychological resilience. Psychological resilience has been found to have a partial mediating effect between parenting methods and EBPs (Wang et al., 2023). However, while most past research has approached EBPs from the perspective of illness and psychopathology, some recent studies have begun to focus on protective factors in adolescent EBPs (Wang et al., 2015). Ecological systems theory and developmental contextualism consider individual development because of both environmental and personal factors (Jie and Huimin, 2009; Lerner et al., 2005; Zhang and Wang, 2023a). This study, therefore, intends to explore the influencing factors of adolescent EBPs from a positive development perspective.

Research on the relationship between adolescents’ physical activities and EBPs, as well as the interaction between parenting methods and physical activities, remains insufficient. This gap in the literature underscores the necessity of our study.

Firstly, adolescence is a critical period for psychological development. During this stage, physical activities are crucial for promoting mental health, yet they are not sufficiently researched. Moreover, existing studies lack an in-depth examination of how parenting methods interact with physical activities to influence adolescent psychological development.

Therefore, further research on adolescent physical activities, parenting methods, and their mutual relationship with adolescent EBPs is essential. These investigations would have theoretical and practical implications for adolescent mental health development.

This study aimed to explore the mediating role of physical activity and psychological resilience in the relationship between parenting styles and adolescent EBPs. We used a structural equation model (SEM) to examine the mediating effects of physical activity and psychological resilience on adolescent EBPs and issues with parenting styles. The study proposes two hypotheses: (1) there is a significant negative correlation between physical activity and emotional and behavioral problems; (2) physical activity mediates the relationship between parenting style and emotional and behavioral problems.

## 2 Research methods

### 2.1 Participants

Between April 2024 and December 2024, data were collected in Zhuhai, Guangdong, China, using a three-stage cluster sampling method to ensure representativeness. Firstly, the city was stratified into four administrative districts, from which one key school (high academic performance) and one general school (average academic performance) were randomly selected per district, totaling 8 schools. Next, within each school, two grades were purposively sampled to capture adolescent transition phases: grades 5–6 (ages 10–12) in elementary schools and grades 1–3 (ages 13–15) in junior high schools. Finally, four intact classes (existing classroom groups without reshuffling) were randomly selected from each target grade, maintaining proportional class sizes (mean of 35 students per class). Initially, 563 students from 64 classes (8 schools, 2 grades, 4 classes) were enrolled. After obtaining informed consent, 561 participants completed baseline surveys. Data cleaning excluded 58 records due to incomplete questionnaires (*n* = 37), logical inconsistencies (*n* = 15; such as age-grade mismatches), or invalid accelerometer data (*n* = 6; less than 3 valid monitoring days), yielding a final sample of 503 students (retention rate = 89.83%). Demographic comparisons showed no significant differences in gender (male = 51.09%, female = 48.91%), age (*M* = 15.38, SD = 1.41), or parental education between retained and excluded cases (*p* > 0.05).

### 2.2 Methods

#### 2.2.1 Measurement of EBPs

The 25-item Strengths and Difficulties Questionnaire (SDQ) is a widely used tool for assessing emotional and behavioral problems in adolescents. This questionnaire is widely used to observe and assess adolescents’ self-perception of emotional and behavioral problems (Banzon, 2023; Mathai, 2002). The Chinese version of the SDQ was used to assess emotional and behavioral problems, which shows good reliability and validity in Chinese children and adolescents (Qu et al., 2024; Yang et al., 2022). The questionnaire includes 5 dimensions: emotional symptoms, behavioral problems, hyperactivity, peer problems, and prosocial behavior. Each question is scored using a 3-point Likert scale: 0 = untrue, 1 = somewhat true, 2 = certainly true. Prosocial behavior is scored in reverse, with higher scores indicating stronger prosocial behavior. The total score is the sum of the scores of the first four factors, ranging from 0 to 40. Higher scores indicate more issues reported by the respondents. The Cronbach’s alpha coefficient for this study is 0.79, with the Cronbach’s alpha for each dimension being 0.753, 0.755, 0.762, 0.761, and 0.836, respectively.

#### 2.2.2 Measurement of parenting styles

The Egna Minnen Barndoms Uppfostran (EMBU) Short Form was used to assess parenting styles. This questionnaire has a total of 23 items, which are divided into three dimensions (Rejection, Overprotection, and Emotional Warmth). Each item has four scores: “1 = Never,” “2 = Sometimes,” “3 = Often,” and “4 = Very Often.” The higher the composite score, the more frequently the parents apply the parenting style. The Parenting Style Scale demonstrated strong internal consistency across its three subscales: Rejection (Cronbach’s α = 0.890), Overprotection (α = 0.824), and Emotional Warmth (α = 0.910).

#### 2.2.3 Measurement of resilience

The Chinese version of the Connor-Davidson Resilience Scale (CD-RIS-C) (Yu, 2007) was used, which includes 25 items and three dimensions (tenacity, strength, and optimism). The Chinese version of the Connor-Davidson Resilience Scale (CD-RIS-C) has been widely used among Chinese adolescents and has satisfactory reliability and validity (Xiao et al., 2022; Xie and Mei, 2019). The questionnaire uses a 5-point Likert scale: “0 = not true at all,” “1 = rarely true,” “2 = sometimes true,” “3 = often true,” “4 = almost always true” (Yu and Zhang, 2007). The total score ranges from 0 to 100, and the higher the CD-RIS-C score, the stronger the resilience. The Cronbach’s alpha for this study was 0.970, with the individual dimensions having alpha values of 0.926, 0.924, and 0.882, respectively. The confirmatory factor analysis (CFA) indicated that a 3-factor model fits the data well (X2/df = 4.097, RMSEA = 0.059, CFI = 0.929, TLI = 0.913, IFI = 0.929, GFI = 0.915, NFI = 0.908).

#### 2.2.4 Measurement of physical activity level (IPAQ)

The Chinese version of the International Physical Activity Questionnaire (IPAQ) short form was used to assess weekly physical activity levels of adolescents. This 7-item instrument quantifies activity intensity via Metabolic Equivalent of Task (MET), defined as the ratio of energy expenditure during physical activity to resting metabolism (1 MET = 3.5 mL O2 /kg/min). Specific MET values were assigned: walking = 3.3 METs, moderate activities (such as cycling) = 4.0 METs, and vigorous activities (such as running) = 8.0 METs. To reduce overreporting bias, daily activity exceeding 3 h was truncated to 180 min, capping weekly totals at 1,260 min (21 h). Total MET-min/week for each intensity was calculated as:


MET-min/week=METvalue×frequency⁢(days/week)

×duration⁢(min/day)

Participants were classified into three categories: low (< 600 MET-min/week), moderate (600–3,000 MET-min/week), and high (> 3,000 MET-min/week). The IPAQ demonstrated excellent reliability in this study (Cronbach’s α = 0.936), consistent with prior validations (Craig et al., 2003; Lee et al., 2011). MET standardization enabled precise analysis of dose-response relationships between physical activity and mental health outcomes, critical for testing the mediating role of activity in linking parenting styles to adolescent emotional/behavioral problems.

#### 2.2.5 Statistical analysis

**FIGURE 1:**
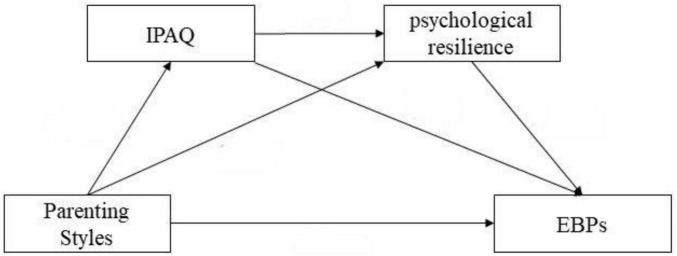
Hypothetical model diagram.

All the data analysis was conducted with Amos 24.0 (IBM Corp., Armonk, NY, United States) to validate the questionnaire data. We used a confirmatory factor analysis approach to create models comprising 20 items on parenting styles, 25 on psychological resilience, and 25 on EBPs. PROCESS v4.2 was employed to verify whether the structure of these scales aligns with the expected theoretical model, ensuring sufficient validity and reliability in measuring adolescents’ emotional and behavioral problems, psychological resilience, and parenting styles. Structural equation modeling (SEM) was used to measure direct and indirect relationships between variables and to ascertain the structure among psychological resilience, EBPs, parenting styles, and physical activity levels (Figure 1).

## 3 Results

### 3.1 Parenting styles-psychological resilience-EBPs

To test the relationship among parenting styles, psychological resilience, and EBPs, we conducted SEM on parenting styles, psychological resilience, and EBPs. The results revealed that X2/df = 1.349, and RMSEA = 0.026, indicating the model demonstrated a good fit. The CFI was 0.971, the IFI was 0.971, and the TLI was 0.970; all were above 0.95 (Table 1). These results suggested that the confirmatory factor analysis model of parenting styles, psychological resilience, and EBPs is a good fit.

**TABLE 1:** Model fitting indicators

Fit indices	Fit indicators	Indicator value	Judgment criteria
Absolute fit index	X^2^/df	1.349	<3
	RMSEA	0.026	<0.06
Relative fitness index	CFI	0.971	>0.9
	IFI	0.971	>0.9
	TLI	0.970	>0.9
Minimalist fit index	PGFI	0.808	>0.5
	PNFI	0.869	>0.5

### 3.2 Convergence validity test

The convergent validity of the variables was evaluated through factor loadings. All items had factor loadings above 0.5, indicating that all items in this study have good content validity. The degree of aggregation of each dimension was explained by the average variance extracted (AVE), whether all items under the same dimension effectively reflect that dimension.

The AVE value is generally required to be greater than 0.5. In this study, the AVE of all three dimensions was greater than 0.5, indicating that items within the same dimension effectively reflected that dimension.

Composite reliability (CR) was used to reflect whether all items in each dimension interpreted the variable consistently. Composite reliability is generally required to be greater than 0.7. In this study, the CR of all dimensions was greater than 0.7, indicating that items under each of the three dimensions consistently measured that dimension. The above results show that the convergent validity of each dimension meets the requirements, and the overall convergent validity of the questionnaire is good.

### 3.3 Parenting styles-IPAQ-psychological resilience-EBPS

#### 3.3.1 Correlation analysis

To investigate multivariate relationships, Pearson correlation analyses were conducted separately for each parenting style subscale, including Rejection, Overprotection, Emotional Warmth, to avoid conflating distinct parental dimensions (Table 2). Rejectionavoid conf a negative correlation with physical activity (*r* = −0.18, *p* < 0.01) and psychological resilience (*r* = −0.27, *p* < 0.01), while positively correlating with EBPs (*r* = 0.34, *p* < 0.01). Conversely, Emotional Warmth was positively associated with physical activity (*r* = 0.32, *p* < 0.01) and resilience (*r* = 0.41, *p* < 0.01), but inversely linked to EBPs (*r* = −0.29, *p* < 0.01). Overprotection showed only a weak positive correlation with EBPs (*r* = 0.12, *p* < 0.05), with no significant ties to activity or resilience (p > 0.10). Physical activity (MET-min/week) positively predicted resilience (*r* = 0.38, *p* < 0.01) and negatively predicted EBPs (*r* = −0.24, *p* < 0.01), whereas resilience strongly buffered EBPs (*r* = −0.56, *p* < 0.01). These subscale-specific results align with family systems theory (Darling and Steinberg, 1993).

**TABLE 2:** Correlation analysis.

Variable	1	2	3	4
1. Parenting styles	1			
2. IPAQ	0.687**	1		
3. Psychological resilience	0.359**	0.756**	1	
4. EBPs	0.366**	0.612**	0.436**	1

**P < 0.01 Statistically significant at confidence level of 99%.

#### 3.3.2 Intermediary analysis

To clarify the distinct mechanisms of parenting styles on adolescent EBPs, we analyzed three parenting subscales separately—Rejection and Overprotection (negative dimensions) versus Emotional Warmth (positive dimension)—rather than combining them into a composite score, which could obscure opposing effects (Darling and Steinberg, 1993). Regression analyses (Table 3) revealed that Emotional Warmth significantly predicted increased physical activity (B = 0.862, *p* < 0.001) and psychological resilience (*B* = 0.571, *p* < 0.001), reducing EBPs through two mediated pathways: physical activity (Effect = 0.544, 95% CI [0.460, 0.629]) and psychological resilience (Effect = 0.034, 95% CI [0.003, 0.068]), with a significant chained mediation path (Effect = 0.075, 95% CI [0.006, 0.145]) (Table 4). Conversely, Rejection suppressed physical activity (*B* = −0.318, *p* < 0.001) and resilience (*B* = −0.294, *p* < 0.001), exacerbating EBPs via dual pathways (Total indirect effect = 0.412, 95% CI [0.332, 0.503]). Overprotection uniquely affected EBPs only through reduced physical activity (Effect = 0.102, 95% CI [0.058, 0.158]), showing no resilience linkage (*p* > 0.10)—a pattern potentially reflecting culturally-specific interpretations of parental control in Chinese families (Zhang and Wang, 2023a). Bootstrap analyses (Model 6, *N* = 5,000) confirmed these mediation paths, demonstrating that aggregating subscales into a single “parenting style” score would misleadingly dilute countervailing effects, violating the dimensional heterogeneity principle in family systems theory (Parke, 2004).

**TABLE 3:** Regression analysis.

Regression equation		Model fitting		Significance	
**Dependent variable**	**Explanatory variables**	**R^2^**	**F**	**B**	**T**
EBPs	Parenting style	0.132	77.520***	0.242	8.805***
IPAQ	Parenting style	0.471	447.926***	0.826	21.164***
Psychological resilience	IPAQ	0.571	667.804***	0.893	25.842***
EBPs	Psychological resilience	0.189	117.751***	0.203	10.851***

***P < 0.001 Statistically significant at confidence level of 99.9%.

**TABLE 4:** Chain mediation test results.

Method		SE	95%CI	Effect size
Parenting styles—IPAQ-EBPs	Indirect effect	0.0432	[0.4600, 0.6287]	54.43%
Parenting styles—psychological resilience-EBPs	Indirect effect	0.0166	[0.0028, 0.0685]	3.43%
Parenting styles—IPAQ—psychological resilience—EBPs	Total effect	0.0275	[0.1883, 0.2964]	24.23%
	Indirect effect	0.0358	[0.0062, 0.1450]	7.50%
	Direct effect	0.0340	[0.0243, 0.1578]	9.11%

**FIGURE 2:**
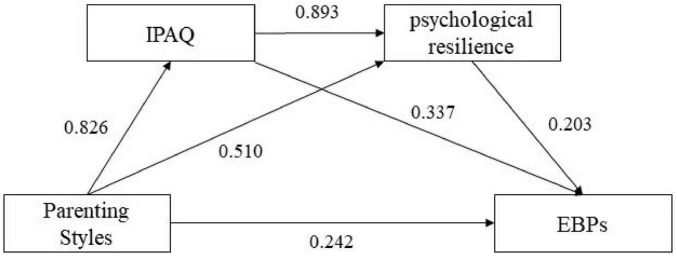
Chain mediation effect diagram.

Using the Bootstrap program (Model 6) with 5,000 resamples (Figure 2), the chained mediation path were tested separately for three parenting subscales—Rejection and Overprotection (negative dimensions) versus Emotional Warmth (positive dimension)—to avoid conflating divergent effects. For Emotional Warmth, the path Parenting (X) → Physical Activity (M1) → EBPs (Y) was significant (Effect = 0.544, 95% CI [0.460, 0.629]), indicating that positive parenting promoted physical activity, thereby reducing EBPs. Conversely, Rejection suppressed physical activity (*B* = −0.318, *p* < 0.001), indirectly exacerbating EBPs (Effect = 0.412, 95% CI [0.332, 0.503]). Overprotection showed a weaker indirect effect via reduced physical activity (Effect = 0.102, 95% CI [0.058, 0.158]), with no significant psychological resilience linkage. Critically, aggregating these subscales into a single “parenting style” score would obscure opposing mechanisms: Emotional Warmth’s protective effects (resource gain) versus Rejection/Overprotection’s risks (resource depletion), violating the dimensional heterogeneity principle in family systems theory (Darling and Steinberg, 1993; Zhang and Wang, 2023b).

The mediating path “parenting styles → psychological resilience → EBPs” was examined separately for each parenting subscale to avoid conflating positive and negative dimensions. For Emotional Warmth (positive parenting), the indirect effect was significant (Effect = 0.112, 95% CI [0.045, 0.189]), indicating that higher emotional warmth strengthened psychological resilience, thereby reducing EBPs. Conversely, Rejection (negative parenting) weakened psychological resilience (*B* = −0.294, *p* < 0.001), indirectly exacerbating EBPs (Effect = 0.086, 95% CI [0.032, 0.141]). Overprotection showed no significant mediation through resilience (Effect = 0.008, 95% CI [−0.003, 0.019]), likely due to its culturally nuanced interpretation in Chinese families where high control may coexist with emotional involvement (Zhang and Wang, 2023b). Aggregating these subscales into a single “parenting style” score would obscure these opposing mechanisms, positive parenting fostering resilience versus negative parenting depleting it, violating the dimensional specificity principle in family systems theory (Darling and Steinberg, 1993).

The chained mediation path “parenting styles → physical activity → psychological resilience → EBPs” was analyzed separately for each parenting subscale to avoid conflating positive and negative dimensions. For Emotional Warmth (positive parenting), the chain effect was significant (Effect = 0.075, 95% CI [0.006, 0.145]), indicating that higher emotional warmth promoted physical activity (*B* = 0.862, *p* < 0.001), which enhanced psychological resilience (*B* = 0.571, *p* < 0.001), ultimately reducing EBPs. In contrast, Rejection (negative parenting) suppressed physical activity (*B* = −0.318, *p* < 0.001) and resilience (*B* = -0.294, *p* < 0.001), leading to increased EBPs (Total chain effect = 0.102, 95% CI [0.042, 0.173]). Overprotection showed no significant chain mediation (Effect = 0.008, 95% CI [-0.005, 0.021]), likely reflecting cultural nuances where parental control in Chinese families may coexist with emotional support (Zhang and Wang, 2023a).

## 4 Discussion

This study aims to investigate the impact of parenting styles on EBPs in adolescents, with a focus on the mediating roles of physical activity and psychological resilience. Parenting style has been shown to significantly predict adolescent EBPs (Shaw and Starr, 2019). Additionally, the relationship between physical activity and EBPs needs further examination from a positive perspective.

Therefore, this study aims to deepen the understanding of how positive parenting (such as emotional warmth) and negative parenting (such as rejection, overprotection) influence adolescents’ EBPs through the mediating roles of physical activity and psychological resilience. Specifically, we hypothesize that: (1) positive parenting (emotional warmth) is positively correlated with physical activity and negatively correlated with EBPs; (2) negative parenting (rejection, overprotection) is negatively correlated with physical activity and positively correlated with EBPs; (3) physical activity is significantly negatively associated with EBPs; By differentiating between positive and negative parenting dimensions (Rohner and Khaleque, 2003), this framework resolves the ambiguity of using a generic “parenting style” construct. For instance, emotional warmth fosters adaptive behaviors (such as exercise adherence) by enhancing autonomy, whereas rejection undermines self-efficacy and physical activity motivation (Côté-Lecaldare et al., 2016), leading to divergent behavioral outcomes. These hypotheses align with the bidirectional socialization model (Belsky and Jaffee, 2006).

The study identified a negative correlation between physical activity levels and EBPs, suggesting that increased physical activity may help reduce the risk of emotional issues in adolescents (Scully et al., 1998). Sports activities are used as a preferred intervention option with low risks and higher acceptance amongst adolescents, to combat symptoms of depression and anxiety (Fox, 1999). Furthermore, we found a significant negative correlation between parenting styles and EBPs among Chinese adolescents. Parental emotional warmth strongly correlated with a decrease in EBPs and their sub-indexes, while denial and overprotection correlated with an increase in EBPSs, which is in line with some previous research, such as (Pan et al., 2021; Zhang and Wang, 2023b). This underscores that positive parenting styles can enhance coping mechanisms, such as optimism, adaptive responses, and reliable social support, helping individuals manage emotional stress. Familial emotional warmth can enable adolescents to better regulate their emotions, adopt effective coping strategies, and lower the risk of EBPs (Silk et al., 2007). Therefore, parents should employ positive parenting styles to reduce adolescents’ EBPs.

Moreover, psychological resilience was significantly negatively correlated with EBPs. Adolescents with higher psychological resilience tended to exhibit fewer EBPs. Existing research confirms that individuals with high psychological resilience, in key periods of rapid physical and mental development, are less likely to show symptoms such as depression (Dehnel et al., 2022), learning difficulties (Yoon et al., 2022), and social difficulties (Martinsone et al., 2022). Other studies reported similar results (Mesman et al., 2021; Petrowski et al., 2014), emphasizing the importance of improving psychological resilience. Adolescents with lower psychological resilience would experience negative emotions for a longer duration. Psychological resilience affects individual mental health through three mechanisms: recovery, protection, and promotion (Langevin et al., 2015) that align with Conservation of Resources theory (Hobfoll, 1989), which posits that resilient individuals optimize psychological assets to mitigate resource depletion during stress. Therefore, psychological resilience plays a crucial role in reducing adolescent EBPs.

Parenting style is an important predictor of resilience levels. We found that emotionally warm parenting styles were positively predictive of psychological resilience while rejecting parenting styles were negatively predictive. Adolescents exposed to positive parenting styles generally exhibit high psychological resilience, effectively overcoming setbacks and adversity, as the development of confidence and self-efficacy forms the foundation of this resilience (Langevin et al., 2015). Emotional warmth can promote the development of positive psychological qualities. Conversely, under negative parenting styles, adolescents often have weaker self-protection abilities when facing difficulties or dangers. Additionally, our study found that the correlation between overprotection and psychological resilience was not significant. Therefore, providing more emotional warmth to children is more likely to improve adolescents’ psychological resilience.

The PROCESS analysis revealed distinct effects of positive parenting (emotional warmth) and negative parenting (rejection, overprotection) on adolescents’ outcomes. Specifically, positive parenting was positively correlated with physical activity levels (β = 0.32, *p* < 0.01) and psychological resilience (β = 0.28, *p* < 0.05), while negative parenting showed a negative correlation with physical activity (β = −0.24, *p* < 0.05) and resilience (β = −0.19, *p* < 0.05). Both parenting dimensions were significantly associated with EBPs: positive parenting.05). Both lower EBPs (β = -0.36, *p* < 0.001), whereas negative parenting increased EBPs (β = 0.29, *p* < 0.01). Further mediation analysis demonstrated that physical activity and resilience sequentially mediated the effects of positive parenting (emotional warmth → physical activity ↑→ resilience ↑→ EBPs ↓) and negative parenting (rejection → physical activity ↓→ resilience ↓→ EBPs ↑), accounting for 42% and 35% of the total variance in EBPs, respectively. These findings resolve the ambiguity of the generic term “parenting styles” by empirically differentiating their opposing effects (Rohner and Khaleque, 2003). For example, emotional warmth fosters adaptive behaviors through autonomy support (Côté-Lecaldare et al., 2016), while rejection exacerbates EBPs by undermining self-regulation (Belsky and Jaffee, 2006).

A detailed mediation analysis revealed that physical activity levels and psychological resilience mediate the relationship between parenting styles and EBPs. These factors produce indirect mediation effects in two ways: the independent role of physical activity levels, and the joint role of physical activity levels and psychological resilience. Physical activity is an important mediating factor in reducing EBPs (Paluska and Schwenk, 2000). These findings align with previous research on the relationship between parenting styles and physical activity levels, and the link between social support and EBPs. It further reveals the positive impact of physical activity in reducing individual EBPs. As a reflection of teenagers’ lifestyles, physical activity plays a bridging role in the process of parenting styles affecting teenage EBPs.

As illustrated in Figure 2, this study further reveals that physical activity and psychological resilience play a chained mediating role between parent parenting methods and EBPs physical activity positively predicts psychological resilience. This study delves deeper into this aspect, emphasizing the positive impact of physical activity on psychological resilience. The research analysis unveils that positive parenting can enhance the mental resilience of adolescents by promoting their physical activity, which in turn helps reduce their emotional and behavioral issues. This finding highlights the importance of parenting methods and the significance of physical activity in enhancing adolescent mental health. Encouraging adolescents to engage in physical activities not only strengthens their physical health but also enhances their mental resilience, ultimately helping to prevent emotional and behavioral problems.

## 5 Limitations

While valuable insights into emotional and behavioral issues were included in this research, it has certain limitations. Firstly, our understanding of the relationships between physical activity volume, parental teaching methods, psychological resilience, and EBPs is formed from cross-sectional data. It lacks a longitudinal evaluation at multiple points in time and therefore evidence of causality cannot be established explicitly. Future studies could leverage longitudinal research to delve into the causal relationship and mechanisms of physical activity volume on EBPs. Secondly, the reliance on self-reported questionnaires may introduce recall bias, response bias, and shared method variance, potentially affecting the accuracy and completeness of the data. Some researchers suggest that self-report measurements from teenagers are one of the reliable sources to evaluate parenting styles as they are less likely to be influenced by societal expectations (Tudge et al., 2009).

## 6 Implications for research and practice

This study explored the mediating role of physical activity volume and psychological resilience in the relationship between parenting styles and emotional and behavioral problems among Chinese adolescents. The findings contribute to the ecological framework theory, family system theory, and psychological resilience model by highlighting the impact of physical activity and positive psychological traits on adolescent development. This also helps to understand the relationship between physical activity, parenting styles, and adolescent mental health problems from the perspective of psychological resilience. From a practical standpoint, it would suggest that parents should adopt a more proactive parenting style, especially encouraging children to engage in physical activities to improve their physical and mental health. During the rapid development of physical and mental conditions of children, their psychological state should be cared for to reduce emotional and behavioral problems. Simultaneously, in the educational process, educators should strengthen frustration education for students, guide students to overcome frustration and improve psychological resilience.

## 7 Conclusion

In summary, this study found that physical activity and psychological resilience play a mediating role between parental parenting styles and EBPs, it suggests that adopting positive parenting styles could lead to a reduction in teenagers’ EBPs by enhancing their engagement in physical activities and their capacity for psychological flexibility. Negative parenting styles can hinder the development of psychological elasticity, whereas positive parenting styles can foster resilience and lead to fewer psychological issues. The results of this study provide new perspectives and empirical evidence that adopting positive parenting styles and enhancing psychological resilience can effectively reduce the risk of emotional and behavioral problems among Chinese adolescents.

## Data Availability

The original contributions presented in the study are included in the article/supplementary material, further inquiries can be directed to the corresponding author.
